# Association of Advisory Committee Votes With US Food and Drug Administration Decision-Making on Prescription Drugs, 2010-2021

**DOI:** 10.1001/jamahealthforum.2023.1718

**Published:** 2023-07-07

**Authors:** C. Joseph Ross Daval, Theodore W. Teng, Massimiliano Russo, Aaron S. Kesselheim

**Affiliations:** 1 Program on Regulation, Therapeutics, and Law, Division of Pharmacoepidemiology and Pharmacoeconomics, Department of Medicine, Brigham and Women’s Hospital and Harvard Medical School, Boston, Massachusetts

## Abstract

**Importance:**

The US Food and Drug Administration (FDA) often relies on independent advisory committees when making decisions about the approval of prescription drugs or their withdrawal from the market. These committees provide the FDA with valuable insight and an opportunity to build public trust through transparent deliberation, but recent controversies have raised questions about the optimal use of FDA advisory committees.

**Objective:**

To assess the frequency, purposes, and voting outcomes of human drug advisory committees convened from 2010 to 2021 and the FDA’s corresponding actions.

**Design, Setting, and Participants:**

This qualitative study used a manual review of meeting summaries prepared by FDA staff for the 18 human drug advisory committees active at any time from January 1, 2010, to December 31, 2021, as well as FDA announcements and press releases, drug labels and approval data, industry publications, and company press releases.

**Main Outcomes and Measures:**

Outcomes of votes on regulatory questions were recorded using meeting minutes. Alignment of FDA action with advisory votes for new drugs and indications was judged as of 1 year after the vote was held and as of November 30, 2022.

**Results:**

The FDA held 409 human drug advisory committee meetings from 2010 to 2021. Committees were convened less frequently over time, from a high of 50 in 2012 to a low of 18 in 2020 and 2021. Much of this decrease occurred at committee meetings involving votes on initial approvals, which declined from a high of 26 in 2012 to a low of 8 in 2021. Overall, FDA regulatory actions aligned with 262 of 298 advisory committee votes on initial approvals, supplemental approvals, withdrawals of approval, and safety actions (88%). Approval followed 142 of 147 positive votes for initial approvals (97%) and 33 of 36 positive votes for supplemental indications (92%), while nonapproval followed 40 of 60 negative votes on initial approvals (67%) and 18 of 21 negative votes on supplemental indications (86%).

**Conclusions and Relevance:**

In this qualitative study, there was consistent alignment between advisory votes and FDA action across years and subject areas, but the number of meetings decreased over time. Discordance between FDA actions and advisory committee votes was most frequently an approval after a negative vote. This study demonstrated that these committees have played a key role in the FDA’s decision-making process but that the FDA sought independent expert advice less frequently over time even as it continued to follow it. The role of advisory committees in the current regulatory landscape should be more clearly and publicly defined.

## Key Points

**Question:**

What were the frequency, purposes, and voting outcomes of US Food and Drug Administration (FDA) human drug advisory committees convened from 2010 to 2021, and what were the FDA’s corresponding actions?

**Findings:**

In this qualitative study of 409 advisory committee meetings, overall, 88% of FDA regulatory actions aligned with advisory committee votes, including votes on initial drug approvals, supplemental approvals, and safety actions. Committees were convened less frequently over time.

**Meaning:**

This study showed that the FDA sought independent expert advice less frequently over time even as it continued to follow it.

## Teaser

This quality improvement study assesses the frequency, purposes, and voting outcomes of US Food and Drug Administration (FDA) human drug advisory committee meetings from 2010 to 2021 and how often the FDA acted in accordance with committee recommendations.

## Introduction

The US Food and Drug Administration (FDA) is responsible for approval and oversight of prescription drugs in the US. Before making regulatory decisions about these products, such as whether to approve a new drug or change its labeling, the FDA can convene advisory committees—independent panels of medical and scientific experts unaffiliated with the FDA as well as patient, community, and industry representatives.^1^ When advisory committees are convened, they usually offer recommendations in the form of a vote.^2^ Although the advice is nonbinding,^3^ the FDA tends to follow it. One analysis of votes from 2008 to 2015 found that the FDA acted against advisory committee advice only 22% of the time.^4^ Another study found 28% overall discordance in new drug approval decisions from 1997 to 2006.^5^

In addition to providing FDA leaders with valuable insight, advisory committees provide an opportunity for the FDA to build and maintain public trust.^6^ When its decisions align with advisory committee recommendations, the FDA uses supportive committee votes in public statements to bolster its credibility, such as when it authorized the COVID-19 vaccine for children^7^ and took steps to withdraw the drug hydroxyprogesterone caproate (Makena).^8^ By law, advisory committee meetings must be open to the public.^9^ This provides a window into how the FDA performs its essential functions and even allows sharing of clinical trial results that would otherwise remain confidential.^10^ Many meetings also include opportunities for commentary from people not on the committee.^11^

Advisory committees’ ability to support the FDA’s credibility may be growing in importance, as polls since the COVID-19 pandemic have found decreasing trust in the FDA as an institution.^12^ Recent controversies, however, have drawn public scrutiny to the FDA’s use of advisory committees. Most notably, the fallout over the FDA’s approval of the Alzheimer disease treatment aducanumab (Aduhelm) despite the near-unanimous negative vote of the advisory committee highlighted the potential reputational harm of overruling advisors without adequate justification.^13,14^ The question of how the FDA chooses whether to convene an advisory committee arose after it declined to reconvene its vaccine advisory committee before authorizing a third dose of the BNT162b2 COVID-19 vaccine (Pfizer-BioNTech) for the general population after that committee had declined to endorse that authorization a few months earlier.^15^ A previous study of negative FDA advisory committee votes found that the proportion of new drugs reviewed by an advisory committee before approval declined dramatically from over half in 2010 to 6% in 2021.^16^ A separate recent analysis found 86% alignment with advisory committee recommendations on 133 approval decisions from 2017 to 2022 but did not address the frequency of meetings or the timing of decisions.^17^

In light of growing concerns about the FDA’s use of advisory committees, we reviewed all advisory committees from 2010 to 2021 related to prescription drug regulation with the aim of assessing how often advisors were convened, for what purposes, and how often the FDA acted in accordance with advisory committee recommendations.

## Methods

In this qualitative study, we collected data on the frequency, purposes, and voting outcomes of human drug advisory committees convened from January 1, 2010, to December 31, 2021, as well as the FDA’s corresponding action. This study was exempt from institutional review board approval and the need for informed consent in accordance with 45 CFR §46.102 because all information was public and no patient-level data were analyzed. The Standards for Reporting of Diagnostic Accuracy (STARD) reporting guideline was followed.^18^

### Meeting Collection

Meeting minutes are summaries prepared by the FDA that state the meeting’s purpose, voting and discussion questions, and outcomes of votes. These were collected from the FDA website for the 18 human drug advisory committees active at any time from 2010 to 2021. Meetings were excluded if they were not held due to cancellation or postponement (n = 31). Two meetings relating to the Controlled Substances Act were also excluded because they involved regulatory actions by the US Drug Enforcement Administration, not the FDA.

If a committee was asked to discuss 2 drugs in 1 day, such as in separate morning and afternoon sessions, the sessions were counted as 2 meetings occurring on the same day, so that each recommendation could be counted separately. If a meeting spanned multiple days, we counted the first day as the meeting date. One multiday meeting from 2021 included 6 distinct votes on whether different indications granted under the accelerated approval pathway should be withdrawn from 3 drugs. This was counted as 6 separate advisory committee meetings to fairly account for each vote.

### Meeting Characteristics

Meeting minutes were reviewed, and meetings were categorized by whether the committee voted on an initial approval, a supplemental indication, the withdrawal of a drug or indication granted accelerated approval, a safety action, or a different regulatory action or did not vote on a regulatory action. This categorization was consistent with methods used in prior studies^4,16^ and did not require double-coding, as it involved minimal subjective judgment.

Initial approval was defined as a question that directly considered approval of a new drug, using language such as “safety and efficacy” or “risk-benefit profile” or questions such as, “Does the evidence support approval?” or “Should approval be delayed?” The initial approval category included all New Drug Applications and Biologics License Applications.

Supplemental indication was defined as a question that directly considered approval of an additional indication for an approved drug, using language such as “favorable risk-benefit profile.” This category included all supplemental New Drug Applications and supplemental Biologics License Applications, as well as questions considering whether to grant an over-the-counter indication for an already approved drug.

Safety action was defined as a question that directly considered an affirmative safety action for an approved drug, including withdrawal of a drug or indication, addition of a contraindication or warning to the drug label, or implementation of a risk evaluation and mitigation strategy (REMS), using language such as “Should a REMS be imposed?” or “Should the product be removed?”

### Recommendations and Corresponding FDA Action

Outcomes of votes on regulatory questions were recorded using meeting minutes. The majority outcome of the vote was recorded as the recommendation of the committee. Corresponding FDA actions were determined using FDA announcements and press releases, drug labels and approvals at Drugs@FDA, the REMS database, and internet searches for industry publications and company press releases.

For meetings considering initial approval, overall approval questions using language such as “risk-benefit” or questions such as “Should it be approved?” were prioritized if available over questions asking about safety or efficacy separately. In meetings with multiple votes, a single question for which the committee voted in favor of an indication, approval for any use, or safety action was considered sufficient to be considered a positive vote.

### Statistical Analysis

Alignment of FDA action with advisory votes for new drugs and indications was judged as of 1 year after the vote was held and as of November 30, 2022. Statistical analysis was conducted using R, version 2022.12.0 + 353 (R Project for Statistical Computing). A survival analysis was conducted on initial approval votes. Survival was measured starting from the vote until either approval or censoring at November 30, 2022. Significance was set at 2-sided *P* < .05.

## Results

The FDA held 409 human drug advisory committee meetings from 2010 to 2021. The committee that met the most times was Oncologic Drugs (78 [19%]), followed by Endocrinologic and Metabolic Drugs (44 [11%]) and Anesthetic and Analgesic Drug Products (41 [10%]) (eTable 1 in Supplement 1). Over half of all meetings (219 [54%]) included votes on initial drug approvals, 59 (14%) on supplemental indications, 28 (7%) on safety actions, 8 (2%) on the withdrawal of a drug granted accelerated approval, 11 (3%) on a different regulatory action, and 82 (20%) did not vote on any regulatory action; at 2 meetings (<1%), the topics could not be categorized because the meetings were closed to the public, which is permitted by federal law under exceptional circumstances.^19^ The 11 votes on different regulatory actions included 1 meeting on an emergency use authorization for molnupiravir (9%) and 10 Pharmacy Compounding Advisory Committee votes on whether to add or remove items from a list of drugs subject to large-scale compounding (91%).

In the 82 meetings in which the committee did not vote on any regulatory action, advisors received presentations and discussed topics of relevance to the FDA, such as study design requirements, pediatric development plans for certain drugs, or approval pathways for certain classes of drugs. One standing committee, the Pharmaceutical Science and Clinical Pharmacology Advisory Committee, never voted on a regulatory question in any of its 13 meetings.

**Figure 1.**
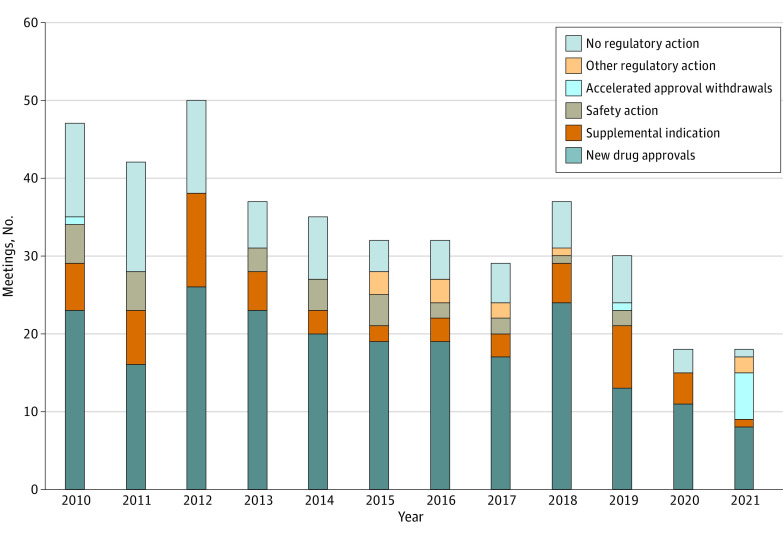
US Food and Drug Administration Drug Advisory Committee Meetings by Topic, 2010-2021

Committees were convened less frequently over time (Figure 1), decreasing from a high of 50 in 2012 to a low of 18 in 2020 and 2021. The largest decline was among committee meetings covering initial approvals, which decreased from a high of 26 in 2012 to a low of 8 in 2021. Although the number of meetings overall decreased, the proportion of meetings involving votes increased, as the number of meetings involving no regulatory vote was 14 in 2011 and 1 in 2021.

### Voting Outcomes

Among the 219 committee meetings with voting on new drug approvals, the committee voted for approval at 149 (68%), the committee voted against approval at 66 (30%), and there was a tied vote at 4 (2%). Among the 59 votes on supplemental indications, 36 (61%) were for approval, 21 (36%) were against approval, 1 (2%) was tied, and 1 (2%) could not be categorized. The 28 safety action votes included 16 votes (57%) on whether a drug label was appropriate, including changes to contraindications, boxed warnings, and dosage instruction changes; 7 votes (25%) on whether to withdraw a drug for safety reasons; 2 votes (7%) on whether to modify REMS (special regulatory requirements placed on drugs with particular safety concerns); 1 vote (4%) on whether to remove a drug from over-the-counter use; and 2 votes (7%) that could not be categorized. Of the 8 votes on whether an accelerated approval drug or indication should be withdrawn, 4 (50%) were for removal and 4 (50%) against removal.

Of the 325 meetings that voted on a regulatory question, questions were addressed for the first time at 317 (98%). Eight committee meetings (2%) included votes on a regulatory question that had already been voted on in a previous meeting. All were repeated votes on the initial approval of a new drug. For 6 of these drugs (cangrelor, dapagliflozin, flibanserin, lorcaserin, mannitol inhalation powder, and phentermine-topiramate), the committee voted against approval in the first meeting and in favor of approval in the second. For the other 2 (buprenorphine with ethylene vinyl acetate and droxidopa), both the initial and repeated vote resulted in positive recommendations. In all 8 cases, the FDA approved the drug after the second meeting.

### Corresponding FDA Actions

**Figure 2.**
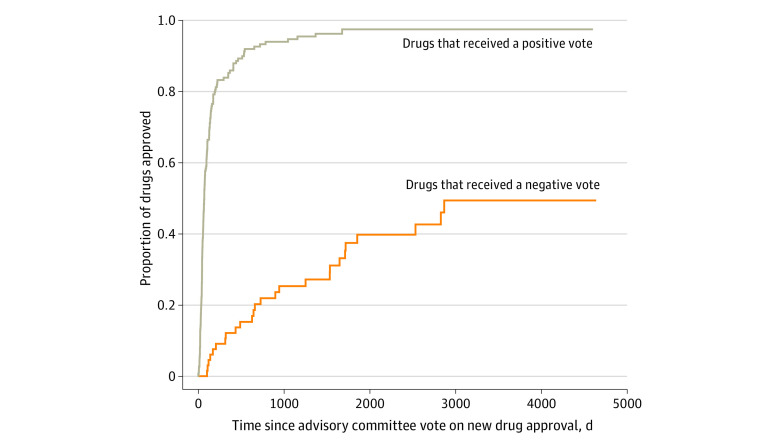
Cumulative Distribution Function of Time to Approval of New Drugs After Advisory Committee Votes, 2010-2021

To assess concordance between advisory committee votes and regulatory actions in the 314 meetings covering initial approval votes, supplemental indication votes, safety action votes, and accelerated approval withdrawal votes, we excluded FDA actions that could not be categorized (3 [1%]), recommendations overridden by later votes (8 [3%]), and ties (5 [2%]) and focused on the remaining 298 meetings (95%). A positive recommendation for an initial approval was associated with a significantly increased likelihood of approval, with a hazard ratio of 9.42 (95% CI, 5.99-14.80; *P* < .001) (Figure 2). Of the drugs that were approved, the median time between a positive vote and approval was 74 days (IQR, 48-147 days) (0.2 years [IQR, 0.1-0.4 years]), while the median time between a negative vote and approval was 700 days (IQR, 295-1567 days) (1.9 years [IQR, 0.8-4.3 years]). The follow-up time from the votes to November 30, 2022, was similar for both groups, with a mean (SD) of 2844 days (1103 days) (7.8 years [3.0 years]) for positive-vote drugs and 2722 days (1317 days) (7.5 years [3.6 years]) for negative-vote drugs.

Measured 1 year after initial approval votes, the FDA had approved 127 of the 149 drugs that had received a positive vote (85%) and declined to approve 58 of the 66 that had received a negative vote (88%), leading to an overall alignment of 185 of 215 (86%). A year after the supplemental indication meetings, the FDA had approved 31 of the 36 indications that had received a positive vote (86%) and declined to approve 18 of the 21 that had received a negative vote (86%), leading to an overall alignment of 49 of 57 (86%).

Measured from November 30, 2022, and excluding the 8 votes that were later overridden, the FDA had approved 142 of the 147 drugs that had received a positive vote (97%) and declined to approve 40 of the 60 that had received a negative vote (67%), leading to overall alignment of 182 of 207 (88%). Measured from November 30, 2022, the FDA had approved 33 of the 36 supplemental indications that had received a positive vote (92%) and had declined to approve 18 of 21 that had received a negative vote (86%), leading to an overall alignment of 51 of 57 (89%).

**Figure 3.**
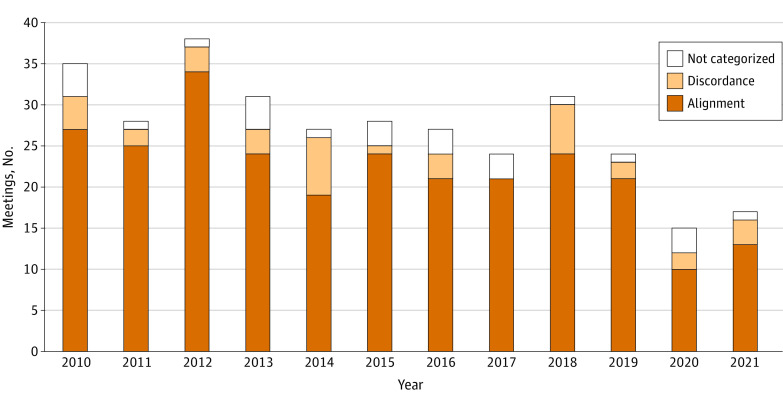
Alignment Between US Food and Drug Administration (FDA) Actions and Advisory Committee Votes Votes not categorized included 5 meetings that resulted in tied votes—4 initial approvals and 1 supplemental indication. The FDA approved 4 of the 5 drugs.

As of November 30, 2022, FDA actions aligned with 262 of 298 advisory committee votes (88%) (Figure 3). Recommendations on initial approvals aligned in 182 of 207 cases (88%), supplemental approvals in 51 of 57 (89%), safety actions in 23 of 26 (88%), and accelerated approval withdrawals in 6 of 8 (75%). The rate at which FDA action aligned with advisors’ recommendations was consistent over time, with the lowest agreement occurring in 2014 for overall regulatory questions (19 of 26 [73%]) and for initial approvals (14 of 19 [74%]).

Negative votes were associated with lower rates of approval for new drugs and supplemental indications. Approval followed 142 of 147 positive votes for initial approvals (97%) and 33 of 36 positive votes for supplemental indications (92%). Nonapproval followed 40 of 60 negative votes on initial approvals (67%) and 18 of 21 negative votes on supplemental indications (86%).

### Discordant Votes

Twenty-five of 207 votes on initial approvals were discordant (12%). Twenty votes were followed by an initial approval (80%), including for hydrocodone bitartrate, paroxetine mesylate, and nebivolol-valsartan. Five drugs were never approved despite a positive vote, including volanesorsen solution, which a majority of the Endocrinologic and Metabolic Drugs Advisory Committee supported (by a vote of 12 to 8) in 2018.

Of the 6 discordant supplemental indication votes, 3 (50%) were approved after a negative vote, including bupivacaine for nerve block. Three (50%) were never approved after a positive vote, including miglustat for Niemann-Pick disease type C.

In the 3 discordant safety recommendations, the FDA declined to take the recommended action. These included votes in favor of contraindications for epidural corticosteroid injection administered to reduce inflammation for pain management, changes to dosage instructions for acetaminophen, and withdrawing epinephrine and racepinephrine delivered via nebulizer from over-the-counter use. The 2 discordant accelerated approval withdrawal votes occurred when the FDA requested withdrawal of 2 accelerated approval cancer indications for atezolizumab after the committee voted against their withdrawal.

Some committees that had negative votes advised waiting for the results of clinical trials based on how the FDA’s voting question was framed. For 2 drugs and 1 additional indication, advisors voted that approval should “be delayed” (selinexor, olaparib) or “be deferred” (pembrolizumab) pending the results of ongoing or prospective clinical trials. For pembrolizumab’s supplemental indication for triple-negative breast cancer, the FDA granted the indication after the results of the trial became available, although it did not reconvene the advisors to consider the new results and advise on the decision.^20^ By contrast, selinexor and olaparib were both granted initial approval before the results of the trial became available against the recommendation of the advisors.^21,22^

Discordance was not concentrated in any year, regulatory decision, or committee. The year with the lowest alignment was 2014 (19 of 26 decisions [73%]), and the category of regulatory decision with lowest alignment was accelerated approval withdrawals (6 of 8 decisions [75%]). The committee with lowest alignment was the Nonprescription Drugs Advisory Committee (3 of 7 decisions [43%]), which made up a low proportion of overall discordance due to the low number of meetings. All 10 committees with the most meetings had alignment of 78% or higher (eTable 2 in Supplement 1).

## Discussion

This study found that FDA regulatory actions were associated with its advisory committee recommendations across new drug approvals, initial indications, and safety actions from 2010 to 2021. The number of total advisory meetings, including for initial approvals, declined by over half between 2010 and 2021. The FDA more often approved drugs after a negative vote than declined to approve drugs after a positive vote, and it rarely reconvened advisors before approval of drugs that had previously received a negative vote.

The consistent alignment between advisory votes and FDA action across years and subject areas suggests that these committees play a key role in the FDA’s decision-making process. This aligns with previous research on the influence of advisory committees on FDA drug approvals.^23,24,25,26^ Explanations for the high level of concordance include that the FDA is often convinced by its advisors’ recommendations or that advisors accurately predict decisions the FDA was already going to make. Another possibility is that the FDA organizes the advisory committee in a way to favor a decision that it wants to make.^27^

The dramatic decrease in meetings suggests that the FDA is leaning away from independent expert advice even as it continues to follow it. It may be that the FDA is becoming more selective about how it convenes committees. Advisory meetings take substantial time and effort, and the cost of preparations could be a factor in the overall decline.^28^ It is also possible that the FDA is relying more on other sources of information, such as its own internal reviews or input from industry experts, to inform its decisions. The COVID-19 pandemic may also have contributed by disrupting FDA operations, although this explanation is limited by the relative ease of replacing in-person with virtual meetings, which the FDA has used since the start of the pandemic. Regardless of the reasons for the decline in meetings, the role of these committees in the current regulatory landscape should be more clearly and publicly defined.

We found that discordance between FDA actions and advisory committee votes was most likely to take the form of FDA approval after a negative vote. Many of the negative-vote approvals occurred years after the vote, when new evidence may have emerged in support of approval after the advisors cast their votes. For this reason, not all instances of discordance were necessarily equivalent to overruling the advisory committee. To account for the availability of evidence over time, previous research on FDA advisory committees in a different period limited follow-up to 1 year, finding that from 2008 to 2015, the FDA was more likely to act restrictively than its advisors when they disagreed.^4^ This conclusion is notable in light of our finding that for initial approvals, alignment after 1 year with positive recommendations was similar to alignment with negative recommendations (85% and 88%). We additionally found that as of November 30, 2022, alignment with positive votes had increased (to 97%) and alignment with negative votes had decreased (to 67%) as the FDA continued to approve drugs from both groups. Because the FDA usually declined to reconvene advisors before approval, the committees that had voted against approval were unable to advise on the new data before the FDA’s approval decision in most cases.

### Limitations

This study is limited in that votes on questions of initial approval often did not include language of specific indications, so we were unable to systematically compare approved indications with advisory recommendations for initial approvals. Second, although most voting panelists are experts, some represent patients or other consumer or industry interests. We did not account for how nonexpert panelists with voting authority may have impacted recommendations. Third, the wording of key voting questions asking the same regulatory question, such as approval, varied substantially, potentially influencing advisor votes. How wording of voting questions influences outcomes is a valuable topic for future research.

## Conclusions

In this qualitative study of FDA advisory committees, recommendations were associated with FDA regulatory actions on prescription drugs, with the FDA acting concordantly with its advisors’ recommendations in a majority of cases and over time. However, a decline was observed from 2010 to 2021 in the number of committees that were consulted and asked to vote on these issues. In cases of discordance, the FDA was more likely to favor approval than were its advisors, and the FDA declined to reconvene advisors before most approvals of drugs that had received a negative vote. Reforms are necessary to clarify the FDA’s commitment to independent expert advice as an integral part of its regulation of prescription drugs.

## Notes

Supplement 1. **eTable 1.** US Food and Drug Administration Advisory Committee Meetings, 2010-2021 **eTable 2.** Alignment of Advisory Committee Votes With US Food and Drug Administration Regulatory Action, 2010-2021

Supplement 2.
Data Sharing Statement
