# Supplementary material for: Association of Advisory Committee Votes With US Food and Drug Administration Decision-Making on Prescription Drugs, 2010-2021
Source: JAMA Health Forum. 2023 Jul 7;4(7):e231718. doi: 10.1001/jamahealthforum.2023.1718 (PMC10329213; doi:10.1001/jamahealthforum.2023.1718)
Supplement: Supplement 1. — eTable 1. US Food and Drug Administration Advisory Committee Meetings, 2010-2021 eTable 2. Alignment of Advisory Committee Votes With US Food and Drug Administration Regulatory Action, 2010-2021 [file jamahealthforum-e231718-s001.pdf]

Supplemental Online Content

Daval CJR, Teng TW, Russo M, Kesselheim AS. Association of advisory committee votes with US Food and Drug Administration decision-making on prescription drugs, 2010-2021. *JAMA Health Forum.* 2023;4(7):e231718. doi:10.1001/jamahealthforum.2023.1718

eTable 1. US Food and Drug Administration Advisory Committee Meetings, 2010-2021

eTable 2. Alignment of Advisory Committee Votes With US Food and Drug Administration Regulatory Action, 2010-2021

© 2023 Daval CJR et al. *JAMA Health Forum.*

Table 1. FDA Advisory Committee Meetings, 2010-2021

		Oncologic Drugs	Endocrin-ologic and Metabolic Drugs	Anesthetic and Analgesic Drug Products	Antimi-crobial Drugs	Cardio-vascular and Renal Drugs	Arthritis	Obstetrics, Reproductive and Urologic Drugs	Pulmonary-Allergy Drugs	Psychophar-macologic Drugs	Gastro-intestinal Drugs	Peripheral and Central Nervous System Drugs
	Total	78	44	41	35	30	24	23	21	19	17	16
2010	47	6	8	4	2	7	3	2	3	1	3	4
2011	43	9	3	1	3	2	1	3	2	1	5	4
2012	50	10	7	2	6	4	4	2	1	0	3	1
2013	37	6	6	0	2	2	3	4	4	1	1	2
2014	36	4	3	3	4	8	1	3	3	1	0	0
2015	32	5	6	3	2	1	1	1	5	1	0	1
2016	32	3	3	7	2	0	3	2	0	5	1	1
2017	29	10	2	4	0	0	2	1	0	2	0	1
2018	37	3	2	10	7	0	2	2	1	3	4	1
2019	30	8	3	1	5	1	2	3	1	1	0	0
2020	18	5	0	6	0	3	0	0	1	2	0	1
2021	18	9	1	0	2	2	2	0	0	1	0	0

© 2023 Daval CJR et al. *JAMA Health Forum.*

		Pharmaceutical Science and Clinical Pharmacology	Dermatologic and Opthalmic Drugs	Nonprescription Drugs	Pharmacy Compounding	Antiviral Drugs	Drug Safety and Risk Management	Medical Imaging Drugs
	Total	13	12	10	10	8	5	3
2010	47	3	0	0	0	1	0	0
2011	43	3	2	1	0	3	0	0
2012	50	3	3	1	0	2	1	0
2013	37	1	0	1	0	2	1	1
2014	36	0	1	4	0	0	1	0
2015	32	0	3	0	3	0	0	0
2016	32	0	1	1	3	0	0	0
2017	29	1	1	1	2	0	0	2
2018	37	1	0	0	1	0	0	0
2019	30	1	1	1	0	0	2	0
2020	18	0	0	0	0	0	0	0
2021	18	0	0	0	1	0	0	0

© 2023 Daval CJR et al. *JAMA Health Forum.*

Table 2. Alignment of Advisory Committee Votes with FDA Regulatory Action, 2010-2021

	**All Advisory Committees**	Oncologic Drugs	Endocrinologic and Metabolic Drugs	Anesthetic and Analgesic Drug Products	Antimicrobial Drugs	Cardiovascular and Renal Drugs	Arthritis	Obstetrics, Reproductive and Urologic Drugs	Pulmonary-Allergy Drugs
**Combined Regulatory Actions**	**298**	**56**	**37**	**26**	**28**	**23**	**20**	**18**	**19**
Concordant	**262**	**47**	**34**	**21**	**26**	**21**	**20**	**14**	**17**
Alignment	0.88	0.84	0.92	0.81	0.93	0.91	1	0.78	0.89
**Initial Approvals**	207	37	24	20	23	18	12	12	14
Concordant	182	31	22	17	21	16	12	8	12
Alignment	0.88	0.84	0.92	0.85	0.91	0.89	1	0.67	0.86
**Supplemental Indication Approvals**	57	12	8	3	3	4	6	0	4
Concordant	51	11	7	2	3	4	6	0	4
Alignment	0.89	0.92	0.88	0.67	1	1	1		1
**Safety Actions**	26	0	5	3	2	1	2	5	1
Concordant	23	0	5	2	2	1	2	5	1
Alignment	0.88		1	0.67	1	1	1	1	1
**AA Withdrawals**	8	7	0	0	0	0	0	1	0
Concordant	6	5	0	0	0	0	0	1	0
Alignment	0.75	0.71						1	

© 2023 Daval CJR et al. *JAMA Health Forum.*

	Psychopharmacologic Drugs	Gastrointestinal Drugs	Peripheral and Central Nervous System Drugs	Dermatologic and Opthalmic Drugs	Nonprescription Drugs	Antiviral Drugs	Medical Imaging Drugs	Drug Safety and Risk Management
**Combined Regulatory Actions**	**17**	**11**	**15**	**9**	**7**	**7**	**3**	**2**
Concordant	**16**	**11**	**11**	**9**	**3**	**7**	**3**	**2**
Alignment	0.94	1	0.73	1	0.43	1	1	1
**Initial Approvals**	13	7	11	8	0	6	2	0
Concordant	13	7	7	8	0	6	2	0
Alignment	1	1	0.64	1		1	1	
**Supplemental Indication Approvals**	2	4	3	1	5	1	0	1
Concordant	1	4	3	1	3	1	0	1
Alignment	0.5	1	1	1	0.6	1		1
**Safety Actions**	2	0	1	0	2	0	1	1
Concordant	2	0	1	0	0	0	1	1
Alignment	1		1		0		1	1
**AA Withdrawals**	0	0	0	0	0	0	0	0
Concordant	0	0	0	0	0	0	0	0
Alignment								

For blank cells, alignment could not be calculated because no meetings were held.

© 2023 Daval CJR et al. *JAMA Health Forum.*
