# Supplementary material for: Association of Advisory Committee Votes With US Food and Drug Administration Decision-Making on Prescription Drugs, 2010-2021
Source: JAMA Health Forum. 2023 Jul 7;4(7):e231718. doi: 10.1001/jamahealthforum.2023.1718 (PMC10329213; doi:10.1001/jamahealthforum.2023.1718)
Supplement: Supplement 2. — Data Sharing Statement [file jamahealthforum-e231718-s002.pdf]

Data Sharing Statement

Daval. Association of Advisory Committee Votes With US Food and Drug Administration Decision-Making on Prescription Drugs, 2010-2021. *JAMA Health Forum*. Published July 07, 2023. doi:10.1001/jamahealthforum.2023.1718

Data
Data available: No

Additional Information
Explanation for why data not available: All relevant data are publicly available online
